# An Energy Saving System for a Beam Pumping Unit

**DOI:** 10.3390/s16050685

**Published:** 2016-05-13

**Authors:** Hongqiang Lv, Jun Liu, Jiuqiang Han, An Jiang

**Affiliations:** 1 School of Electronic and Information Engineering, Xi’an Jiaotong University, Xi’an 710049, China; hongqianglv@mail.xjtu.edu.cn (H.L.); jqhan@mail.xjtu.edu.cn (J.H.); bisgoon@163.com (A.J.); 2 School of Electrical Engineering, Xi’an Jiaotong University, Xi’an 710049, China

**Keywords:** energy saving, beam pumping unit, stroke speed adjustment, balance degree estimation, surface dynamometer card, pump dynamometer card, Internet of Things

## Abstract

Beam pumping units are widely used in the oil production industry, but the energy efficiency of this artificial lift machinery is generally low, especially for the low-production well and high-production well in the later stage. There are a number of ways for energy savings in pumping units, with the periodic adjustment of stroke speed and rectification of balance deviation being two important methods. In the paper, an energy saving system for a beam pumping unit (ESS-BPU) based on the Internet of Things (IoT) was proposed. A total of four types of sensors, including load sensor, angle sensor, voltage sensor, and current sensor, were used to detect the operating conditions of the pumping unit. Data from these sensors was fed into a controller installed in an oilfield to adjust the stroke speed automatically and estimate the degree of balance in real-time. Additionally, remote supervision could be fulfilled using a browser on a computer or smartphone. Furthermore, the data from a practical application was recorded and analyzed, and it can be seen that ESS-BPU is helpful in reducing energy loss caused by unnecessarily high stroke speed and a poor degree of balance.

## 1. Introduction

Beam pumping units have been widely used in the oil production industry, which serves about two-thirds of artificially-lifted oil wells [1,2]. When the natural drive pressure in a well is too low to encourage crude oil to the surface, artificial pumping units can be employed to lift the fluid from the reservoir. Even for the flowing wells, which contain enough energy for crude oil to rise to the surface without any stimulation in the beginning, the natural drive pressure will reduce over time, and an artificial lift process is also needed in the later stage of these oil wells to increase their production life. Therefore, the artificial pumping unit is generally necessary for most oil wells [3]. There are nearly 920,000 oil wells all over the world, and about 87% of them are operated with the help of artificial pumping units [4]. Different types of artificial pumps can be found in oil production fields, such as sucker rod pumps, hydraulic pumps, plunger piston pumps, electrical submersible pumps, and so on. Among them, sucker rod pumping units are well-known and generally understood, which can be further divided into two categories including beam pumping units and non-beam pumping units. The beam pumping unit accounts for approximately 71% of the artificial lift machinery market in the oil production field [4,5].

The energy saving of the beam pumping unit plays an important role in solving the problems of low profit, energy crisis, and environmental pollution during oilfield development. Beam pumping units have a huge installed capacity around the world, and it is responsible for one-third [6] of the total energy consumption in oilfields. Unfortunately, the energy efficiency of this kind of artificial lift machinery is obviously low, although there is a certain degree of difference according to various well conditions. For example, in China, there are at least 100,000 beam pumping units in use, the total installed capacity can reach up to 3500 MW, and the power consumption is approximately more than 10 billion kWh per year [7]. However, the energy efficiency of this artificial lift machinery ranges only from 12% to 23% [8]. It is considered that the energy saving of beam pumping units has a lot of room for improvement and a good application prospect.

There are three common ways in which the energy saving of beam pumping units can be carried out for efficient oil production. Firstly, improvement of the mechanical structure. The mechanical structure of the beam pumping unit is adjusted for energy saving, such as a double-horsehead pumping unit, curved beam pumping unit, front-mounted pumping unit, and other heterotypic pumping units [9,10]. Secondly, replacement of traditional motors. The traditional three-phase asynchronous motor on the beam pumping unit is replaced by a specially-designed motor to improve the energy efficiency, such as high-slip motor [11] and double-fed induction motor [12]. Finally, installation of additional control devices. A novel electronic controller is introduced to adjust the operating state of the beam pumping unit according to the conditions of the oil well for energy saving, such as a motor control device with a power-off control strategy [13] and an energy saving system based on a wavelet neural network [14]. It can be seen that only an additional controller is needed to be installed without any change of the beam pumping unit itself in the third way.

The automatic adjustment of the stroke speed of the beam pumping unit is one of the most effective means of energy saving in the oil production field. For a high-production oil well, it is usually concerned with increasing the stroke speed of the pumping unit as much as possible to improve the efficiency of oil production. However, for low-production wells and high-production wells in the later stage, it is unacceptable that the economic value of the oil that is pumped from the reservoir to the surface in each stroke is not greater than the cost of the consumed electricity. Some strategies have been adopted to solve this problem. A novel power-off control strategy was proposed to serve for the energy saving of the pumping unit [13] and a dual pulse width-modulation frequency converter was introduced to keep the sucker rod running in an up-slowly-and-down-fast mode [7].

The balance deviation rectification of the beam pumping unit is also important for energy saving. Once a pumping unit is well balanced, it is not only beneficial to safety, but can also convert the excess energy of the motor into the gravitational potential energy of the counterweight on the beam during the downstroke, and could help the motor to lift a heavier load with the conversion of this gravitational potential energy during the following upstroke. On the contrary, if the counterweight of the beam pumping unit is not properly matched, the motor would have to consume more power and convert a part of it into the vibration and noise of the pumping unit. Recently, some new devices and ideas have been proposed to solve this problem, such as an energy feedback device [15] and a redesigned beam pumping unit [16], but these options have a much higher cost compared with the balance deviation rectification of the original pumping unit. In practice, there are several alternative methods that can be used to detect the degree of balance, such as ratios of time, peak current, total power consumption, and peak torque between the downstroke and upstroke of the pumping unit. These methods have high practical value, but the manpower cost is large without the help of an automatic balance degree estimation system.

In this paper, an online energy saving system for beam pumping units (ESS-BPU) was proposed. The stroke speed could be adjusted automatically according to the conditions of oil wells, the degree of balance could be estimated in real-time, and the operating states can be monitored online using a browser on a computer or smartphone so as to make a timely response to an abnormal situation when necessary. The structure of the rest of the paper is organized as follows: Section 2 introduces the principle of this energy saving system, including the overall architecture, design ideas, and necessity of the Internet of Things (IoT); in Section 3, the methods that were used to determine the optimal stroke speed, estimate the degree of balance, and evaluate the performance of pumping unit were explained. Section 4 describes the hardware and software components of the energy saving system. In Section 5, the application of this system in oilfield is discussed, including the installation and result analysis. Finally, the conclusions are given in Section 6.

## 2. Principle of the Energy Saving System

**Figure 1:**
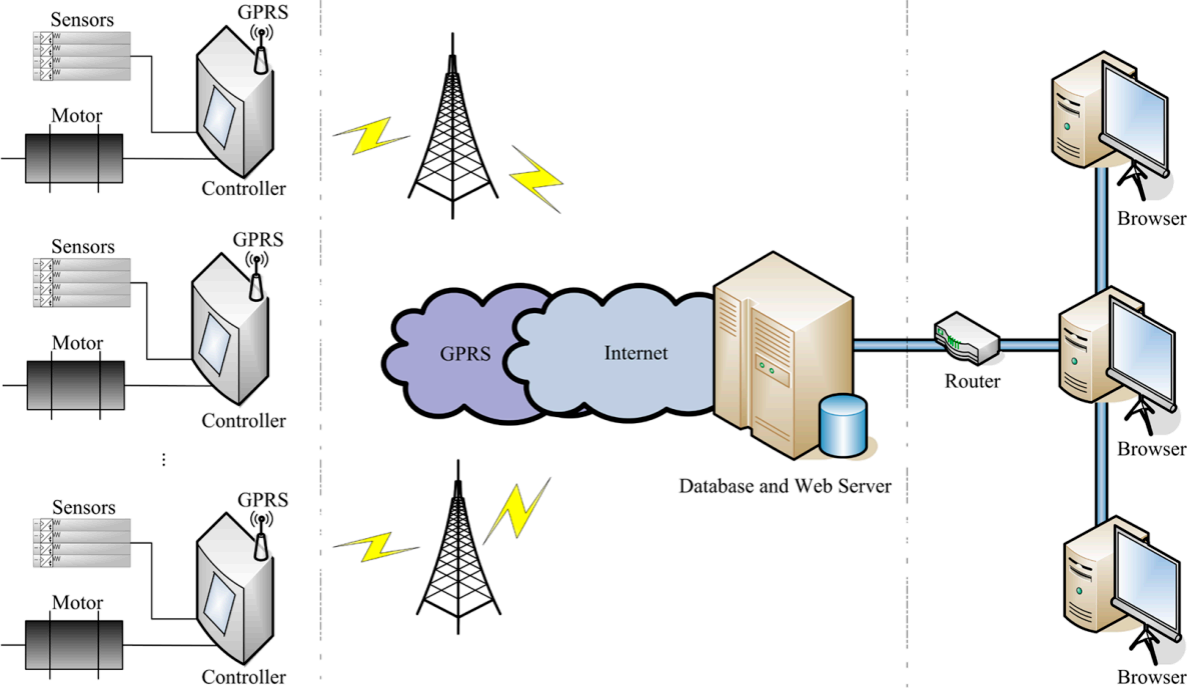
Overall architecture diagram of the energy saving system.

The overall architecture diagram of an ESS-BPU is shown in Figure 1. Efforts have been made from the following three aspects to design the online system for the energy saving of beam pumping unit.

- Automatic adjustment of the stroke speed. A stroke speed determination unit and a motor control unit are combined to carry out the automatic stroke speed adjustment. In the speed determination unit, the load and displacement of the polished rod of the beam pumping unit are measured by a load sensor and an angle sensor, respectively. Then, these two types of data are fed into a wave equation to get a pump dynamometer card, and the optimal stroke speed is determined so as to maximize the area of the pump dynamometer card. In the motor control unit, a frequency converter is used to adjust the motor flexibly to make sure that the pumping unit is running at the optimal stroke speed.
- Estimation of the degree of balance. The input voltage and current of the three-phase motor of the beam pumping unit is measured by a set of voltage and current sensors. Then, the real-time active power of this motor is calculated, and the ratio of the total power consumption between the downstroke and upstroke is regarded as the indicator to assess the balance degree of the pumping unit.
- Remote supervision of the operating states. The remote supervision platform consists of two parts, including a database and web server unit, as well as a remote monitoring unit. The database and web server unit receive the real-time status data sent from different oilfields by means of General Packet Radio Service (GPRS), stores them in the database and delivers web pages to remote terminals. The remote monitoring unit allows clients to monitor the operating states of pumping units using a browser on a computer or smartphone and make a timely response to abnormal situations.

ESS-BPU could be considered as a practical application of IoT in the energy saving of pumping units. The energy saving device in the oilfield and remote supervision platform online are integrated together to serve the energy saving of the pumping unit.

The energy saving device aims to reduce the unnecessary energy consumption by means of automatic stroke speed adjustment and balance degree estimation. A total of four types of sensors are employed to detect the operating conditions of beam pumping unit, including the load and displacement of a polished rod, as well as the input voltage and current of motor. The controller receives data from these sensors, calculates the optimal stroke speed, estimates the degree of balance, and adjusts the motor to make sure that the pumping unit is running at the optimal stroke speed. However, the energy saving efficiency of this device will be greatly reduced while the pumping unit works under abnormal conditions, such as sand production from the oil well, leakage of tubing and paraffin deposits on pipe walls, and the existing device lacks the ability to detect these complex anomalies. Therefore, it is necessary to collect and analyze the operating data manually and periodically.

The remote supervision platform is designed for online monitoring of operating states and timely responses to abnormal situations. Firstly, the pumping unit is often located in an inaccessible area and has a relatively dispersed geographical distribution, this centralized monitoring platform can greatly reduce the frequency of manual inspections over a long distance. More importantly, the information on this platform is conductive to the judgment of abnormal situations and helpful to recovery of the energy-saving efficiency of the device. The real-time, historical, and statistical data hosted in the database server can be accessed through a terminal browser in some kind of meaningful form, which allows clients to make a comprehensive analysis remotely. Thus, it is convenient to identify, or even predict, which kind of abnormal condition the pumping unit is or will be under so that a timely response can be made and the energy-saving efficiency of this device could be recovered as soon as possible.

## 3. Methods

### 3.1. Method of Stroke Speed Determination

The optimal stroke speed is determined with the help of a pump dynamometer card of the beam pumping unit. There are two kinds of common dynamometer cards that can be used for computer diagnosis of the pumping unit, including a surface dynamometer card and a pump dynamometer card [17]. A surface dynamometer card is a curve of load *vs.* displacement at the suspension point of the polished rod throughout a complete stroke cycle, but it is often difficult to understand how the downhole pump is operating from this kind of dynamometer card [18,19]. A pump dynamometer card is a curve of load *vs.* displacement at the downhole pump throughout a complete stroke cycle. It could be employed to diagnose the downhole operating conditions of the pumping unit, including pump performance, tubing problems, leaking pump valves, fluid pound, and so on [20]. The area of the pump dynamometer card is one of the most important methods to determine the applied work of a downhole pump and the fluid yield of the pumping unit [20,21]; that is to say, the optimal stroke speed could be determined so as to maximize the area of the pump dynamometer card.

Several methods have been proposed for the calculation of the pump dynamometer card, one of the most common and effective methods is to solve the boundary value problem of a wave equation given by Gibbs, based on the data corresponding to the surface dynamometer card [22,23]. The technique considers the polished rod as a transmission line, the downhole pump is a transmitter, the suspension point of the polished rod at the surface is a receiver, the load and displacement at the transmitter can be derived from the corresponding signals at the receiver. 

The final solution of this wave equation is as follows:

(1) $$\left\{ \begin{array}{l} U(x,t)=\frac{\sigma_{0}}{2EA}x+\frac{\upsilon_{0}}{2}+\sum_{n=1}^{\bar{n}}\left(O_{n}(x)\cos n\omega t+P_{n}(x)\sin n\omega t\right)\\ F(x,t)=EA\left[\frac{\sigma_{0}}{2EA}+\sum_{n=1}^{\bar{n}}\left(O_{n}^{\prime }(x)\cos n\omega t+P_{n}^{\prime }(x)\sin n\omega t\right)\right] \end{array}\right.$$

where F(x,t) and U(x,t) are the dynamic load and displacement at the depth x down along the polished rod for time t separately, $\sigma_{0}$ and $\upsilon_{0}$ are Fourier coefficients associated with the load and displacement curves measured at the suspension point of the polished rod respectively, E and A are the elastic modulus and cross-sectional area of the polished rod, $\bar{n}$ is the number of truncated Fourier series, $\omega =\frac{2\pi }{T}$ in which T is the period of a complete stroke cycle, and the definitions of $O_{n}(x)$, $P_{n}(x)$, $O_{n}^{\prime }(x)$, and $P_{n}^{\prime }(x)$ can be found in Gibbs’s original paper [22,23].

In this work, the load *vs.* time curve $F(x,t|x=x_{s})$ and displacement *vs.* time curve $U(x,t|x=x_{s})$ at the suspension point of the polished rod were measured by a load sensor and an angle sensor, respectively, and the surface dynamometer card was acquired first. Then, the dynamic model given by Gibbs was employed to transform $F(x,t|x=x_{s})$ and $U(x,t|x=x_{s})$ at the surface to $F(x,t|x=x_{p})$ and $U(x,t|x=x_{p})$ at the downhole pump separately, and the pump dynamometer card was obtained. Finally, the optimal stroke speed was determined to maximize the area of this pump dynamometer card. Here $x_{s}$ denotes the position of the suspension point at the surface, and $x_{p}$ indicates the position at the downhole pump down along the polished rod. In practice, the stroke speed was increased by a specific incremental step, and the speed which can achieve the peak area was ultimately selected as the optimal stroke speed.

### 3.2. Method of Balance Estimation

There are several laws that can be used to estimate the balance degree of beam pumping unit, such as ratios of time, peak current, total power consumption, and peak torque between the downstroke and upstroke. Among these methods, the time and current laws may be easier to implement, but their accuracies are relatively low. The torque law could achieve a higher accuracy, but the measurement of the torque of a motor is difficult. Additionally, the power law may be more practical compared with the other laws.

In the paper, this power law was chosen to evaluate the balance degree of the beam pumping unit. The input voltage U(t) and current I(t) of the motor were measured by a set of voltage and current sensors, then the active power in a stroke cycle was obtained, and the ratio of total power consumption between the downstroke and upstroke was calculated to assess the balance degree of the pumping unit. The ratio λ is defined as:

(2) $$\lambda =\frac{\int_{t\in T_{d}}(U(t)\times I(t)\times \cos\varphi )dt}{\int_{t\in T_{u}}(U(t)\times I(t)\times \cos\varphi )dt}$$

where $T_{d}$ and $T_{u}$ are the half periods of the downstroke and upstroke in a complete stroke cycle, respectively, and $T=T_{d}+T_{u}$. In practice, the degree of balance of the beam pumping unit is considered acceptable when λ∈[0.8,1.0].

### 3.3. Method of Performance Evaluation

Several metrics could be used to evaluate the performance of an energy saving system. In the paper, the active power saving rate $\eta_{a}$, reactive power saving rate $\eta_{r}$, and total energy saving rate $\eta_{t}$ were adopted to quantify the energy saving efficiency of the proposed system. The definitions of these three metrics are as follows:

(3) $$\left\{ \begin{array}{l} \eta_{a}=\frac{W_{1}-W_{2}}{W_{1}}\times 100\\ \eta_{r}=\frac{Q_{1}-Q_{2}}{Q_{1}}\times 100\\ \eta_{t}=\frac{W_{1}-W_{2}+K_{q}(Q_{1}-Q_{2})}{W_{1}+K_{q}Q_{1}}\times 100 \end{array}\right.$$

where $W_{1}$ and $W_{2}$ are the active power consumption for lifting a ton of liquid up one hundred meters before and after the use of the energy saving system, respectively, $Q_{1}$ and $Q_{2}$ are the reactive power consumption for lifting a ton of liquid up one hundred meters before and after the use of this system separately, and $K_{q}$ is the reactive power economic equivalent.

## 4. Composition of the Energy Saving System

ESS-BPU could be regarded as a practical application of IoT technology in energy saving of beam pumping unit. The hardware of ESS-BPU mainly consists of a series of sensors and a controller in oilfield as well as a certain number of computers with different standards online. The software of ESS-BPU can be divided into two parts, including the software embedded in the controller and the supervision platform online.

### 4.1. Hardware

#### 4.1.1. Main Hardware Components

**Figure 2:**
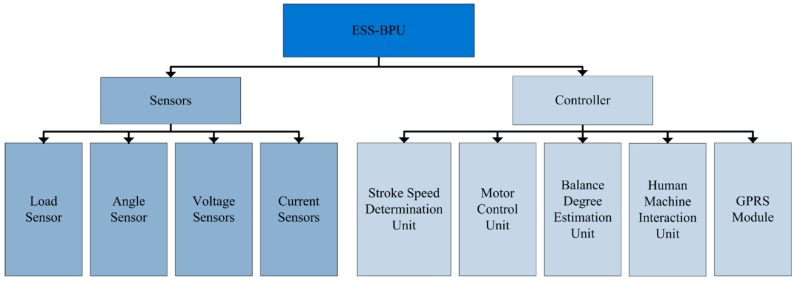
Main hardware components of the energy saving system.

The main hardware components of ESS-BPU are presented in Figure 2. It can be seen that the energy saving device in the oilfield is primarily comprised of four types of sensors and a controller. These sensors include load sensor, angle sensor, voltage sensor, and current sensor. The controller is the center for calculation and control of this energy saving system, which mainly contains a stroke speed determination unit, motor control unit, balance degree estimation unit, human machine interaction unit, and GPRS module.

#### 4.1.2. Sensors

The sensors are employed to detect the operating conditions of the pumping unit automatically and continuously in the oilfield, thus the appropriate ones with certain mechanical and electrical properties should be chosen to satisfy the needs of actual measurement. Table 1 gives some key specifications of the four types sensors involved in this energy system.

It is known that noise usually has a negative impact on the follow-up processing and analysis of signals, especially for the weak analog signal [24]. In this study, the output analog signals of these sensors are often mixed with a variety of noise caused by different kinds of external interference, thus, measures were taken in two ways to reduce this noise before the analog signals arrive at the controller. For each sensor terminal, a closed metal shell was used to shield noises in the process of analog signal amplification. For the analog signal transmission via the wired way, the cables with metal shields were chosen to connect sensor terminals and the controller.

#### 4.1.3. Controller

**Figure 3:**
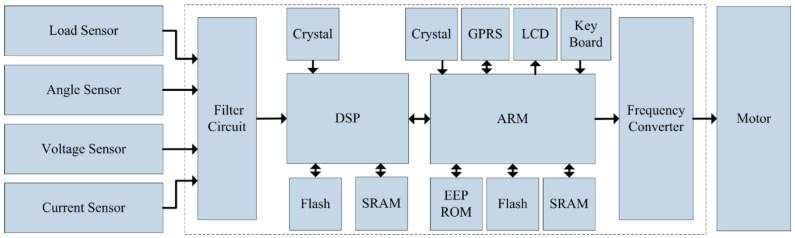
Functional block diagram of the controller.

**Figure 4:**
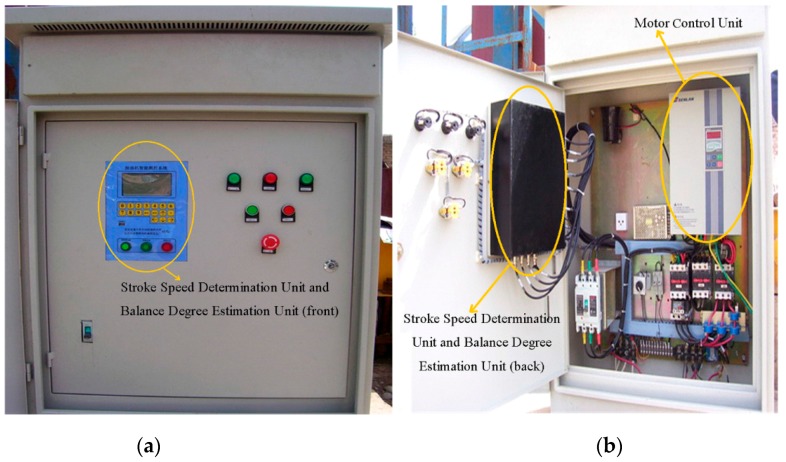
Physical appearance of the controller for stroke speed adjustment and balance estimation. (**a**) Front panel of the controller; and (**b**) inner construction of the controller.

The controller in the oilfield is the core hardware component of the ESS-BPU, which can fulfill the tasks of sensor data receiving, stroke speed adjustment, balance degree estimation, human machine interaction, and data exchange with the sever. Its functional block diagram and physical appearance can be found in Figure 3 and Figure 4, respectively. The following are the main functions and features of this controller:

- Sensor data receiving. Sensor data is the basis of subsequent processing and analysis. A filter circuit was designed to reduce the noises from environment, especially from the frequency converter in this controller, itself. Then the filtered signals were fed into the analog-to-digital converters for digitization.
- Data calculation and motor control. The stroke speed determination unit was dedicated to calculation of the optimal stroke speed by maximizing the area of the pump dynamometer card. The motor control unit was used to adjust the motor speed flexibly by means of a frequency converter to make sure that the pumping unit running at the optimal stroke speed, and the balance degree estimation unit was devoted to the estimation of balance degree of pumping unit. In the controller, a dual-CPU embedded subsystem based on digital signal processor (DSP) and advanced RISC machine (ARM) was designed to implement the tasks of calculation and control, respectively.
- Human machine interaction. A LCD, a key board and several buttons on the front panel were used for the interaction between human and machine. The LCD was employed for the display of menu, real-time status data, curves of dynamometer cards, and so on. The keyboard was mainly responsible for authentication and parameter setting, and the buttons were devoted to manual operation in an emergency, such as system reset, emergency stop, and power switch.
- Data exchange with the server. The network connectivity is essential for a practical application of IoT technology in energy savings of a beam pumping unit. Considering the poor network conditions in an oilfield and the cost of long-range wireless communication, a GPRS module was chosen to implement the real-time data exchange with the server online, instead of a 3G module or even the Internet.

### 4.2. Software

#### 4.2.1. Embedded Software

The software embedded in the controller can be divided into two parts due to its dual-CPU architecture based on DSP and ARM. The software embedded in DSP is mainly used for the digitization of analog signals as well as the calculations of area of pump dynamometer card and ratio of total power consumption between the downstroke and upstroke. The software embedded in ARM is primarily devoted to the overall system task scheduling, such as communication with the DSP, human machine interaction, data exchange with the server under the help of a GPRS module, and motor control by means of a frequency converter. That is to say, the software embedded in the DSP and ARM is generally responsible for the calculation and control tasks separately.

#### 4.2.2. Remote Supervision Platform

The remote supervision platform consists of two parts, including a database and web server unit, as well as a remote monitoring unit. The database and web server unit on the Internet is responsible for receiving, storage, and delivery of the real-time status data of pumping units sent from different oilfields by means of GPRS. The remote monitoring unit allows users to access the data for any specified pumping unit in the platform and make a timely response to abnormal situations.

A browser/server architecture is used in the remote supervision platform, so that the access to this platform and information query could be quite convenient. These operations can be carried out using a browser on a computer or smartphone by any client with appropriate permission. The clients can easily log into the platform via the Internet to approve and register a certain beam pumping unit. Only then can various information of the pumping unit be queried and read, just like any other pumping unit in the platform. There are four kinds of information available from the browser.

- Static information. The information that will not change over a long period of time is declared on the first child page. For example, the location, type, and various mechanical parameters of beam pumping unit, as well as the latest maintenance date, name of the person in charge, contact number, and so on.
- Real-time status. A series of measured values in the latest action cycle can be retrieved from the database server in real-time, including the load and displacement of the polished rod, as well as the input voltage and current of motor. The real-time data is analyzed and processed before being displayed on webpages in an acceptable form, such as curves on a graph or figures in a table. The latest surface dynamometer card, stroke speed, ratio λ, peak/valley load, peak/valley current and other meaningful items can be found on the second child page.
- Historical data. All kinds of status data within the past five days is temporarily retained in the database server, so that the historical status of pumping unit in a valid specified period can be queried and displayed on the historical data query page.
- Statistical results. The optimal operation state since the beginning and statistical results per day are permanently stored in the database server. These data can be used to make a comparative analysis and generate statistical reports of any number of days, and the reports could be printed out, if necessary.

## 5. Results and Discussion

### 5.1. Installation in Oilfields

**Figure 5:**
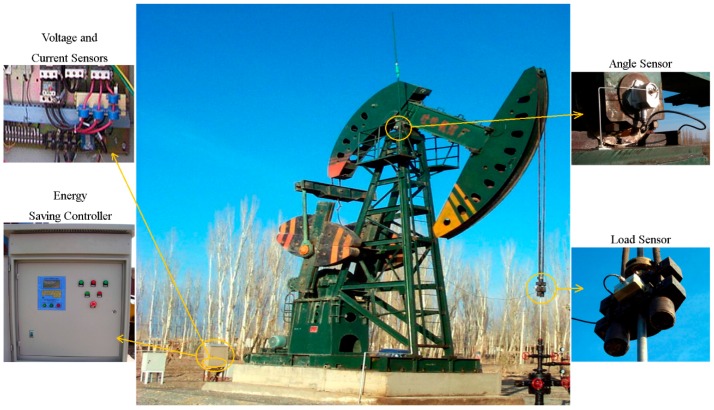
The energy saving device installed on a beam pumping unit, with the actual assembled positions for the four types of sensors and the controller.

ESS-BPUs have been installed and applied in a number of oilfields located in the northwest of China, such as the Huachi oilfield in Gansu province, the Tuha oilfield in Xinjiang province, and the Wuqi oilfield in Shaanxi province. A beam pumping unit and the energy-saving device installed on it are shown in Figure 5. Some important details are magnified, including the actual assembled positions for the four types of sensors and the controller. It can be seen that the load sensor was embedded in the carrier bar of the pumping unit, the angle sensor was fixed at one end of the central rotating shaft of the walking beam, the voltage and current sensors were mounted at the output power lines of the frequency converter, and the controller was employed to replace the original control cabinet for the pumping unit. The sensors measured the operating state of the pumping unit automatically and converted it into analog signals in real-time. The controller received the configuration information entered by the installer, and implemented the tasks of analog signal digitization, calculation, control, and communication with the server online.

### 5.2. Validation of the Utility of ESS-BPU

The actual data for the No. M45 beam pumping unit in the Tuha oilfield was recorded and analyzed in order to validate the utility of this system, including the stroke speed adjustment, balance degree estimation, remote supervision, and efficiency evaluation.

For the automatic adjustment of stroke speed, an incremental stroke frequency strategy was employed to find the optimal stroke speed which can maximize the area of the pump dynamometer card. First of all, the energy saving device installed on the beam pumping unit was configured according to the conditions of oil well and pumping unit. The device was allowed to measure and control once every 20 min, each containing 100 sampling points. The minimum allowable stroke speed was assigned to 3 min^−1^, the maximum allowable stroke speed was set to 10 min^−1^, and the incremental step was chosen as 1 min^−1^. Then the surface dynamometer card was acquired based on the load and displacement data measured at a specific stroke speed, the corresponding pump dynamometer card was obtained by solving the Gibbs’s wave equation, and the optimal stroke speed was finally determined to be 6 min^−1^ so as to the area of the pump dynamometer card could reach the peak value 52,245.02. Table 2 illustrates some of the important points in the process of finding the optimal stroke speed. Since the apply work of downhole pump and the fluid yield of pumping unit could be estimated by the area of the pump dynamometer card, it can be inferred that the device has an ability to avoid energy loss caused by unnecessary high stroke speed while ensuring the liquid yield of the pumping unit. The ability has also been verified in other field tests, however, it was found that this method, which determines the optimal stroke speed so as to maximize the area of the pump dynamometer card, still has some limitations. It cannot be well applied to some special issues, such as sand production from oil wells, leakage of tubing, and paraffin deposit on pipe walls.

For the estimation of the degree of balance, two different states of the beam pumping unit before and after the balance adjustment were compared. The ratio λ given by the energy saving device and the motor power parameters measured by a power testboard under the two states were shown in Table 3. It can be seen that the maximum power, minimum power and average power of the motor when λ=0.92 are all smaller than those when λ=0.65. That is to say, the motor consumes relatively less power while the value of this ratio is within the balance range, and *vice versa*. The comparison result could be indirect evidence of the effectiveness of the ratio in the evaluation of the balance degree of pumping unit.

**Figure 6:**
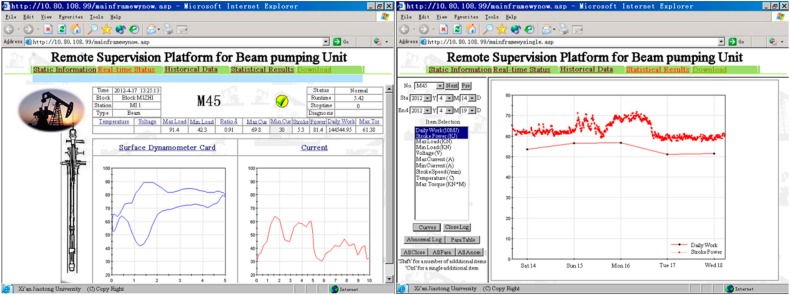
Partial information of the No. M45 beam pumping unit in the remote supervision platform. (**a**) Real-time status; and (**b**) statistics of motor power.

The static information, real-time status, historical data and statistical results of this beam pumping unit can be retrieved from the remote supervision platform. The static information, such as the location, type, and various mechanical parameters, was declared on the first child page. The real-time status, including the surface dynamometer card, stroke speed, ratio λ, peak/valley load, peak/valley current, and other meaningful items for the latest action cycle, were represented on the second child page (Figure 6a). The historical data query page was very similar to this real-time status page except for the extra selection of a past time period. For the statistical results, there are a number of options to choose according to the prevailing concerns, herein the motor power in the past five days were selected to be shown in Figure 6b. It is hoped that the information in this remote supervision platform could be conducive to comparative analysis and timely response.

The overall energy saving efficiency of the proposed system varies with individual well condition, type of pumping unit, and different time periods. In the paper, this efficiency of the system on the No. M45 beam pumping unit was evaluated based on the actual statistical data. The power consumption and liquid yield data over a two-month test period was recorded and compared. The statistical results show that, due to the use of this system, the active and reactive power saving rate of this pumping unit can reach up to 15.45% and 13.83%, respectively, and the average total energy saving rate is 15.32%. It is noteworthy that the power consumption of this system itself, especially the frequency converter, has been taken into account in this efficiency evaluation.

## 6. Conclusions

In this paper, an energy saving system for a beam pumping unit (ESS-BPU) in oil production field was proposed. ESS-BPU has three main functions, including automatic adjustment of the stroke speed, estimation of balance degree and remote supervision online. The real-time status data from a total of four types of sensors is fed into a controller installed in oilfield to implement the tasks of stroke speed adjustment and balance degree estimation, these information is transmitted to a remote supervision platform by means of General Packet Radio Service (GPRS), and clients are allowed to monitor the operating states of pumping unit using a browser on a computer or smartphone so as to make a timely response to abnormal situations when necessary. One of the advantages of this proposed system is the combination of intelligent instrument and Internet of Things (IoT) in the energy saving of beam pumping unit, we can stay in the office, monitor the concerned conditions of pumping unit revealed by this energy saving device from the sensor data, and respond to it only when necessary. In addition, it has been found that several methods have been put forward for automatic regulation of balance and estimation of liquid yield of a beam pumping unit. It is hoped that some of them which can fulfill the actual needs very well could be introduced, so that the energy budget of each pumping unit could be allocated reasonably to optimize the global energy consumption in oilfields.

## Figures and Tables

**Table 1:** Specifications of the four types of sensors.

Parameter	Sensor			
	Load Sensor	Angle Sensor	Voltage Sensor	Current Sensor
Measurement range	0~120 KN	0~360°	0~500 V	0~100 A
Degree of precision	0.95%	0.05°	1.0%	1.0%
Temperature range	−30~70℃	−40~85℃	−40~70℃	−40~70℃

**Table 2:** Results of the automatic stroke speed adjustment using this energy saving system on the No. M45 beam pumping unit.

Stroke Speed	Surface Dynamometer Card	Pump Dynamometer Card	Area of Pump Dynamometer Card
4 min^−1^	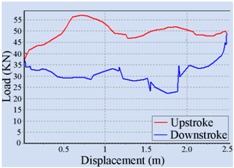	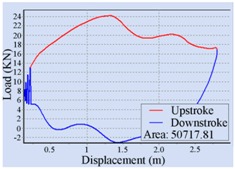	50,717.81
5 min^−1^	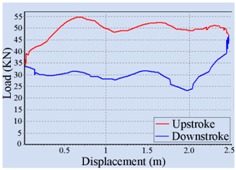	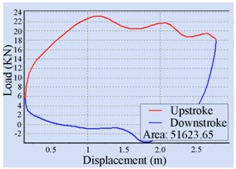	51,623.65
6 min^−1^	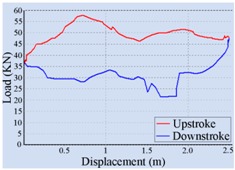	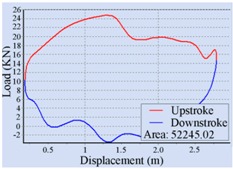	52,245.02
7 min^−1^	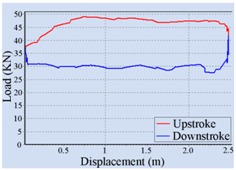	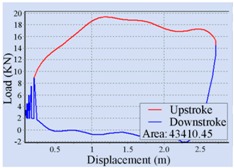	43,410.45
8 min^−1^	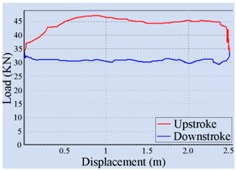	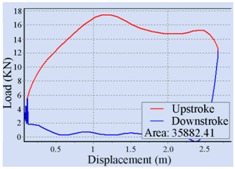	35,882.41

**Table 3:** Balance ratio and the corresponding motor power parameters under two different balance states of the No. M45 beam pumping unit.

Ratio λ	Maximum Power	Minimum Power	Average Active Power	Average Reactive Power
0.65	11.46 KW	−5.88 KW	8.24 KW	5.73 KW
0.92	7.90 KW	−3.14 KW	6.83 KW	4.75 KW
